# Radiation Exposure in Interventional Stroke Treatment: Analysis of the German Neurointerventional Database (DeGIR/DGNR) from 2019 to 2021

**DOI:** 10.1007/s00062-023-01303-0

**Published:** 2023-06-06

**Authors:** Felix Bärenfänger, Peter Schramm, Stefan Rohde

**Affiliations:** 1 Faculty for Health, University of Witten/Herdecke, Alfred-Herrhausen-Straße 50, 58455 Witten, Germany; 2 Department of Medical Physics and Radiation Protection, Klinikum Dortmund gGmbH, Beurhausstr. 40, 44137 Dortmund, Germany; 3 Department of Radiology and Neuroradiology, Campus Lübeck, University Hospital Schleswig-Holstein, Lübeck, Germany; 4 Department of Radiology and Neuroradiology, Klinikum Dortmund gGmbH, Beurhausstr. 40, 44137 Dortmund, Germany

**Keywords:** Dose area product, Diagnostic reference level, Mechanical thrombectomy, Dose dependencies, Dose management

## Abstract

**Purpose:**

To evaluate patient-related radiation exposure in interventional stroke treatment by analyzing data from the German Society for Interventional Radiology and Minimally Invasive Therapy (DeGIR) and the German Society of Neuroradiology (DGNR) quality registry from 2019–2021.

**Methods:**

The DeGIR/DGNR registry is the largest database of radiological interventions in Germany. Since the introduction of the registry in 2012, the participating hospitals have entered clinical and dose-related data on the procedures performed. To evaluate the current diagnostic reference level (DRL) for mechanical thrombectomy (MT) in stroke patients, we analyzed interventional data from 2019 to 2021 with respect to the reported dose area product (DAP) and factors that might contribute to the radiation dose, such as the localization of the occlusion, technical success using the modified treatment in cerebral ischemia (mTICI) score, number of passages, technical approach, additional intracranial/extracranial stenting, and case volume per center.

**Results:**

A total of 41,538 performed MTs from 180 participating hospitals were analyzed. The median DAP for MT was 7337.5 cGy∙cm^2^ and the corresponding interquartile range (IQR) Q_25_ = 4064 cGy∙cm^2^ to Q_75_ = 12,263 cGy∙cm^2^. In addition, we discovered that the dose was significantly influenced by occlusion location, number of passages, case volume per center, recanalization score, and additional stenting.

**Conclusion:**

We conducted a retrospective study on radiation exposure during MT in Germany. Based on the results of more than 41,000 procedures, we observed that the DRL of 14,000 cGy·cm^2^ is currently appropriate but may be lowered over the next years. Furthermore, we identified several factors that contribute to high radiation exposure. This can aid in detecting the cause of an exceeded DRL and optimize the treatment workflow.

## Introduction

Since the publication of the first studies in early 2015 [1] and the meta-analysis of the “Big Five” by Goyal et al. in 2016 [2], mechanical thrombectomy (MT) has become an established procedure for the treatment of acute ischemic stroke. The DAWN and DEFUSE‑3 trials (2018) also demonstrated that MT is superior to intravenous thrombolysis alone, even in patients up to 24 h after symptom onset [3, 4]. Consequently, the number of MTs has increased dramatically in recent years, and further growth is expected in the future [5].

Thus, there is growing interest in monitoring and reducing the resulting radiation exposure for patients and staff during these interventions [6]. In Germany, diagnostic reference levels (DRL) are required by law to limit the patient exposure during radiological procedures (§125 Radiation Protection Ordinance (StrlSchV) and §185 Radiation Protection Act (StrlSchG)). The DRLs were defined by the Federal Office for Radiation Protection (BfS) following the recommendations of ICRP (International Commission on Radiological Protection) 135. The DRLs refer to the mean value of the corresponding parameter over 10–20 (conventional radiography and computer tomography, CT) or 20–30 (fluoroscopy and interventional radiology) studies of the same type [7]. Thus, the DRL may be exceeded in single cases; however, the DRL should not be exceeded by the average value of a study group. The data volume and diversity of centers providing data are crucial for the significance of DRL. For neuroradiological interventions such as MT, the DeGIR/DGNR registry, which was started in 2012, provides the largest dataset and is a representative sample for MT in Germany [8]. The DeGIR/DGNR data were last evaluated in 2018 by Schegerer et al., in which a general DRL of 18,000 cGy∙cm^2^ for the dose area product (DAP) was established for thrombus aspiration [9]. By the end of 2022, the BfS updated the DRL and a generally applicable DRL of 14,000 cGy∙cm^2^ was established for the endovascular treatment of acute stroke [10].

In conventional radiography and CT examinations, the patient dose primarily depends on the patient’s body mass index (BMI) and device settings. The prediction of patient exposure to radiological interventions is considerably more complex, which can lead to large deviations to the DLR in individual cases. Studies have linked these dose deviations to the number of passages [11, 12], occlusion localization [12], and technical approaches [13] during MT; however, owing to the limited number of patients, the results reflect only local centers and a small number of cases. The DeGIR/DGNR data contain a significantly larger number of cases from independent centers and are thus better suited for representing real-world situations when analyzing possible dose dependencies [8].

This study aimed to evaluate the DeGIR/DGNR registry data from 2019–2021 with respect to radiation exposure during MT (expressed by the DAP*)*, including the influence of technical and clinical parameters.

## Methods

### DeGIR/DGNR Registry Data

The DeGIR/DGNR registry data for neurorecanalization (module E, stroke therapy) from 2019–2021 were retrospectively analyzed to evaluate patient exposure during MT.

In addition to DAP and fluoroscopy time (FT) as dose-specific parameters, the DeGIR/DGNR registry provides various demographic and clinical parameters. Table 1 summarizes the parameters used to evaluate possible dose dependence.

**Table 1:** Entries of the DeGIR/DGNR registry data used for the evaluation

Parameter	Input option	Comment
Pharmacological treatment	Yes/No	LIT
Mechanical recanalization	Yes/No	–
Aspiration	Yes/No	–
Stent retrieval	Yes/No	–
Extracranial stenting	Yes/No	Tandem occlusions
Intracranial stenting	Yes/No	–
Occlusion localization	Anterior circulation	only MCA
	Anterior circulation (else)	ACI+ACA/MCA
	Posterior circulation	–
	Multiple territories	–
mTICI score	0	No reperfusion
	1	Contrast agent stasis
	2a	Partial filling < 50% territory
	2b	Partial filling ≥ 50% territory
	2c	Near complete perfusion except slow flow or distal cortical emboli
	3	Complete perfusion
Number of passages	Numeric free text input	–
DAP	Free text input	Unit is entered in additional field
FT	Free text input	Input in minutes

*LIT* local intra-arterial thrombolysis, *MCA* middle cerebral artery, *ACI* internal carotid artery, *ACA* anterior cerebral artery, *MCA* middle cerebral artery, *DAP* dose area product, *FT* fluoroscopy time

### Data Filtration

**Fig. 1:**
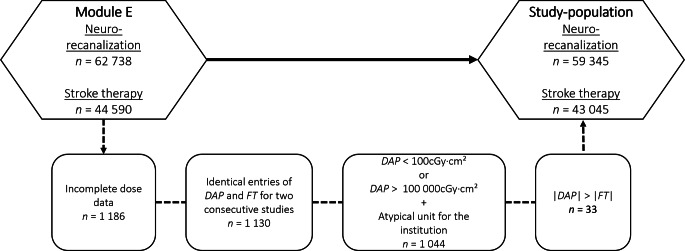
Flowchart for the prefiltering of the DeGIR/DGNR registry data

Participation in the DeGIR/DGNR registry is voluntary, and data can be entered via a web portal (Samedi GmbH, Berlin, Germany). The use of free text fields, for example, for the number of passages, FT or DAP, combined with the large volume of data inevitably leads to incorrect entries. Therefore, non-plausible entries were filtered using a Python data analysis library (pandas) prior to the analysis. The exclusion criteria were incomplete dose data, identical entries of DAP and FT for two consecutive studies, the amount of reported FT exceeding the amount of reported DAP, and unusually high or low DAP values associated with a unit atypical for the institution. The data filtering process is shown as a flowchart in Fig. 1.

### Statistical Methods and Presentation of Results

Descriptive statistics were used to evaluate the results. The mean value (MV) and empirical standard deviation (SD) were calculated to describe the registry data. According to ICRP 135, the results of the dose distributions were described by specifying the median (Q_0.5_) and interquartile range (IQR = Q_0.25_–Q_0.75_). While the median and the first quartile (Q_0.25_) serve as the desired optimization values, the third quartile (Q_0.75_) can be used as a benchmark for establishing diagnostic reference levels [6].

Mood’s median test was used to evaluate significant differences in the medians of more than two sample groups. Dunn’s test was performed to compare individual samples. The general significance level was set at *p* < 0.05. Statistical analyses were performed using OriginPro, version 2022 (OriginLab Corporation, Northampton, MA, USA).

## Results

After applying the filtering criteria (Fig. 1), a total of 59,345 evaluable records remained in Module E of the DeGIR/DGNR registry for 2019–2021. The data were distributed among the following indications: intracranial stenosis (1092), vasospasm therapy (5391), carotid stenting (9817) and stroke treatment (43,045). The details of the study population within the stroke treatment subset are presented in Table 2.

**Table 2:** Study population after application of the filter criteria

Study population *n* = 43,045	
Age (years) MV ± SD (min–max)	74 ± 13.3 (2–106)
Number of centers considered	180
Number of passages MV ± SD (min–max)	2.4 ± 1.8 (1–20)
**Gender**	
Male	20,259 (47.1%)
Female	22,786 (52.9%)
**Technical approach**	
NA	1065
*LIT*	442 (1.1%)
*MT*	41,538 (98.9%)
Aspiration	8290 (21.1%)
Stent retrieval	5805 (14.8%)
Aspiration + stent retrieval	25,221 (64.1%)
**Additional stenting**	
Intracranial stenting	1537 (3.7%)
Extracranial stenting	4221 (10.2%)
Intracranial + extracranial stenting	163 (0.4%)
None	35,617 (85.7%)
**Occlusion localization**	
Anterior circulation (only MCA)	19,953 (48%)
Anterior circulation (else)	16,193 (39%)
Posterior circulation	4378 (10.5%)
Multiple occlusions	1014 (2.5%)
**mTICI score after intervention**	
0	1933 (4.7%)
1	521 (1.3%)
2a	1747 (4.3%)
2b	9733 (23.8%)
2c	3852 (9.4%)
3	23,187 (56.6%)

*MV* mean value, *SD* standard deviation, *LIT* local intra-arterial thrombolysis, *MT* mechanical thrombectomy, *MCA* middle cerebral artery

Analysis of the DeGIR/DGNR registry data for 2019–2021 showed a median of 7337.5 cGy∙cm^2^ and an IQR of 4064–12,263 cGy∙cm^2^ for DAP in MT. The former DRL of 18,000 cGy∙cm^2^ [9], which was valid for the study period, was satisfied in 88% of the cases considered. The DAP exceeded 50,000 cGy∙cm^2^ in 425 (1%) and 100,000 cGy∙cm^2^ in 93 (0.2%) of the cases. In 18 of the 180 participating centers, the third quartile exceeded the former DRL of 18,000 cGy∙cm^2^.

### Recanalization Success

**Fig. 2:**
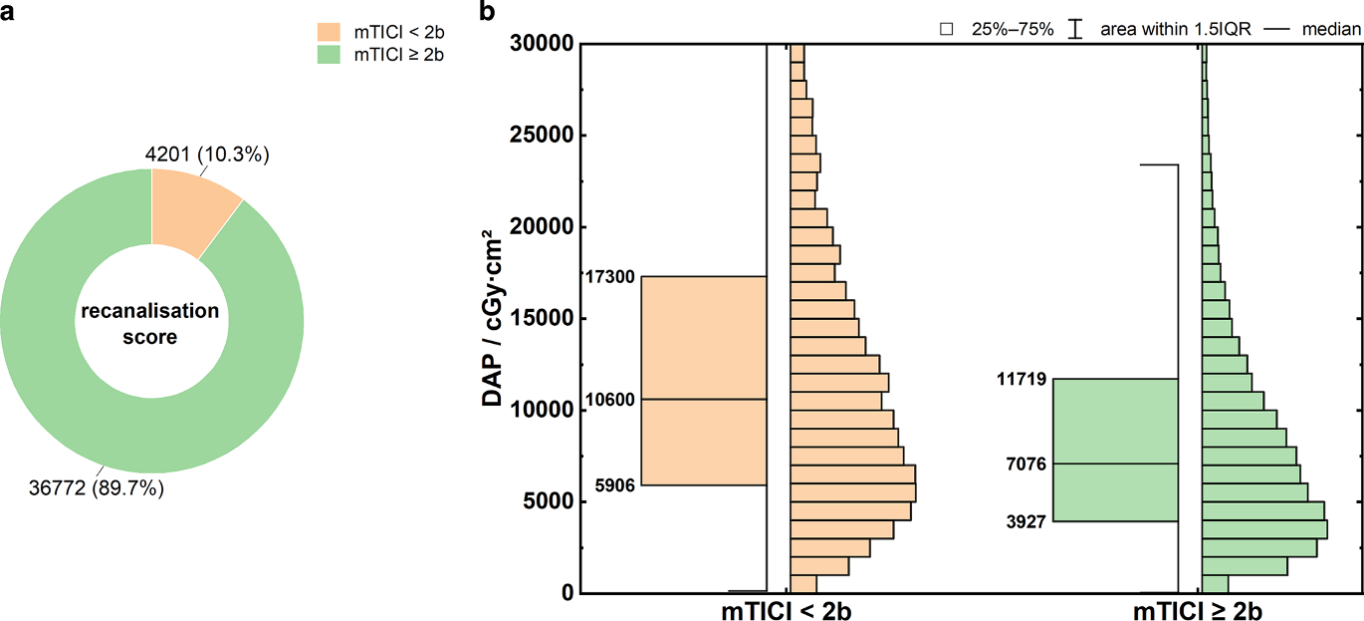
**a** Recanalization rate for MT. **b** Boxplots of DFP distributions as a function of recanalization success. Moodʼs median test: *p* < 0.001. Specification of Q_25_, Q_50_ and Q_75_ at the box plots

Figure 2a shows the distribution of successful recanalization, defined as an mTICI score of ≥ 2b [17]. Thus, 89.7% and 10.3% of the analyzed MTs were successful and unsuccessful, respectively.

Figure 2b shows the DAP distribution as a function of recanalization success. Although no significant differences in patient exposure were observed for mTICI scores of 0–2a (*p* = 0.19), patient exposure decreased with increasing mTICI score of more than 2b (*p* < 0.001). Compared to unsuccessful MT (mTICI < 2b), the DAP was on average approximately 30% lower in patients with successful MT (mTICI ≥ 2b). The key parameters of the distributions are summarized in Table 3, rows 1–2.

**Table 3:** Key parameters of the evaluated DAP distributions. MT includes both MT alone and MT + LIT, and stent retrieval includes stent retrieval alone and stent retrieval + aspiration

	Reference	Data volume	DAP/cGy·cm^2^		
			Q_50_	Q_25_	Q_75_
Recanalization success	mTCI < 2b	4201	10,600	5906	17,300
	mTCI ≥ 2b	36,772	7076	3927	11,719
Technical approach	MT (all)	41,538	7337.5	4064	12,263
	Stent retrieval (with and without additional aspiration)	31,026	7984.5	4538	13,021
	Aspiration only	8290	5280	2880	9140
Additional stenting	None	35,617	6870	3816	11,435
	Intracranial stenting	1537	12,582	8060	20,383
	Extracranial stenting	4221	10,000	6071	16,170
	Intracranial + extracranial stenting	163	17,578	9884	24,393
Occlusion localization	MCA	19,953	6243	3494	10,461
	Anterior circulation (else)	16,193	8460	4820	13,763
	Posterior circulation	4378	8199.5	4417	13,945
	Multiple territories	1014	9900	5400	16,696
Number of passages	1	16,718	5186	2984	8857
	2–3	16,458	7982.5	4719	12,600
	4–7	7152	11,378	7104	17,859
	≥ 8	844	16,600	10,571.5	26,076
Case volume per center/3 years	≤ 150	5678	6570.5	3551	11,370
	151–450	20,912	6839	3641	11,288.5
	≥ 451	14,948	8394	4868	14,061.5

### Technical Approach

**Fig. 3:**
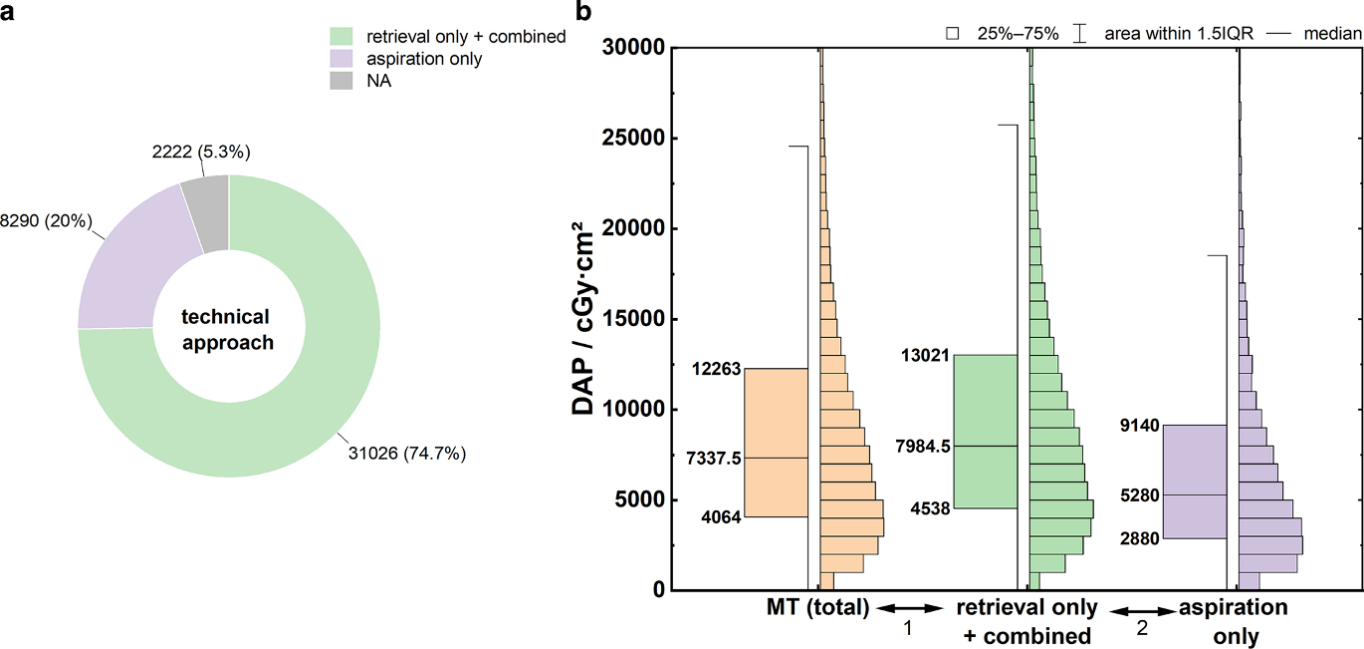
**a** Frequency distributions of the recanalization procedures used in MT. **b** Boxplots of DAP distributions as a function of the recanalization technique. Mood’s median test: *p* < 0.001, Dunn’s test: *p* < 0.001 (1), *p* < 0.001 (2). *MT* mechanical thrombectomy. Specification of Q_25_, Q_50_ and Q_75_ at the box plots

Figure 3a shows the frequency of recanalization techniques used in MT. A distinction was made between stent retrieval (72.1%) and aspiration only (19.3%). For stent retrieval, cases with stent retrieval alone (5805) and the combination technique of stent retrieval + aspiration (25,221) were considered.

Figure 3b shows the dose distributions for *DAP *as a function of the recanalization techniques considered. The key parameters of the distributions are summarized in Table 3, rows 3–5. Radiation exposure was significantly higher when using stent retrieval compared to that using aspiration alone (*p* < 0.001). No significant difference was found between the distribution of stent retrieval alone and aspiration + stent retrieval. When recanalization was achieved solely by local intra-arterial thrombolysis (LIT), the median DAP was only 5700 cGy·cm^2^ (IQR = 3051–9786 cGy·cm^2^) and thus significantly lower than MT (*p* < 0.001).

### Additional Stenting (Extracranial/Intracranial)

**Fig. 4:**
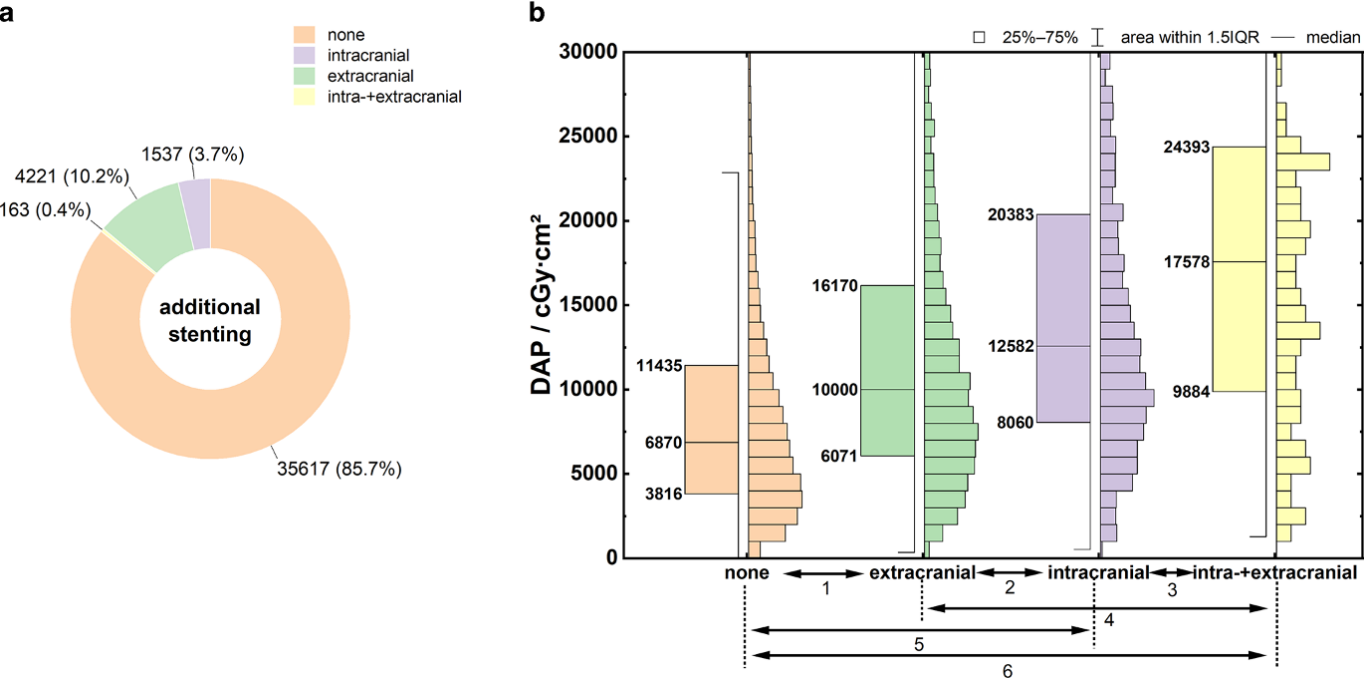
**a** Frequency distribution of additional stenting in MT. **b** Boxplots of DAP distributions as a function of additional stenting. Mood’s median test: *p* < 0.001, Dunn’s test: *p* < 0.001 (1), *p* < 0.001 (2), *p* < 0.06 (3), *p* = 0.001 (4), *p* < 0.001 (5), *p* < 0.001 (6). Specification of Q_25_, Q_50_, and Q_75_ at the box plots

Figure 4a shows the frequency distribution of additional procedures during MT. Extracranial stenting during MT (tandem occlusions), intracranial stenting, and both extracranial and intracranial stenting were required in 10.2%, 3.7%, and 0.4% of the cases, respectively.

Figure 4b shows the resulting dose distributions for the DAP. The key parameters of the distributions are summarized in Table 3, rows 6–9. The need for intracranial and/or extracranial stenting significantly increases the radiation dose, and the highest values are observed with intracranial stenting and the combination of intracranial and extracranial stenting (*p* < 0.001).

### Occlusion Localization

**Fig. 5:**
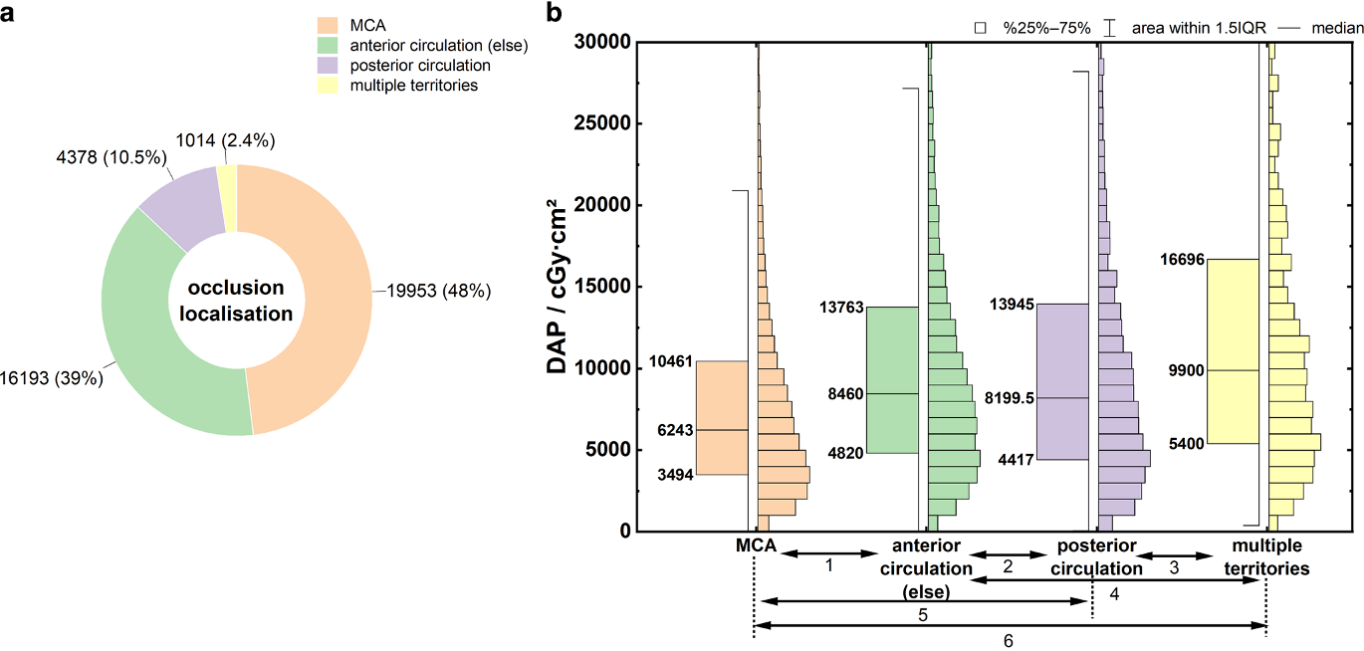
**a** Frequency distributions of the reported occlusion localizations at MT. **b** Boxplots of DAP distributions as a function of occlusion location. Mood’s median test: *p* < 0.001, Dunn’s test: *p* < 0.001 (1), *p* = 0.07 (2), *p* < 0.001 (3), *p* < 0.001 (4),* p* < 0.001 (5), *p* < 0.001 (6). Specification of Q_25_, Q_50_, and Q_75_ at the box plots

Figure 5a shows the frequency distribution of the reported occlusion localizations. Most occlusions (87%) involved the anterior circulation (48% in the middle cerebral artery (MCA), 39% in other vessels), 10.5% involved the posterior circulation, and 2.4% spread over multiple vascular territories.

Figure 5b shows the dose distributions for *DAP* as a function of the occlusion location. The key parameters of the distributions are summarized in Table 3, rows 10–13. The lowest and highest DAP values were observed in occlusions of the MCA (anterior circulation) and multiple vascular territories, respectively. The impact of the location of the occlusion on DAP was statistically significant (*p* < 0.001).

### Number of Passages

**Fig. 6:**
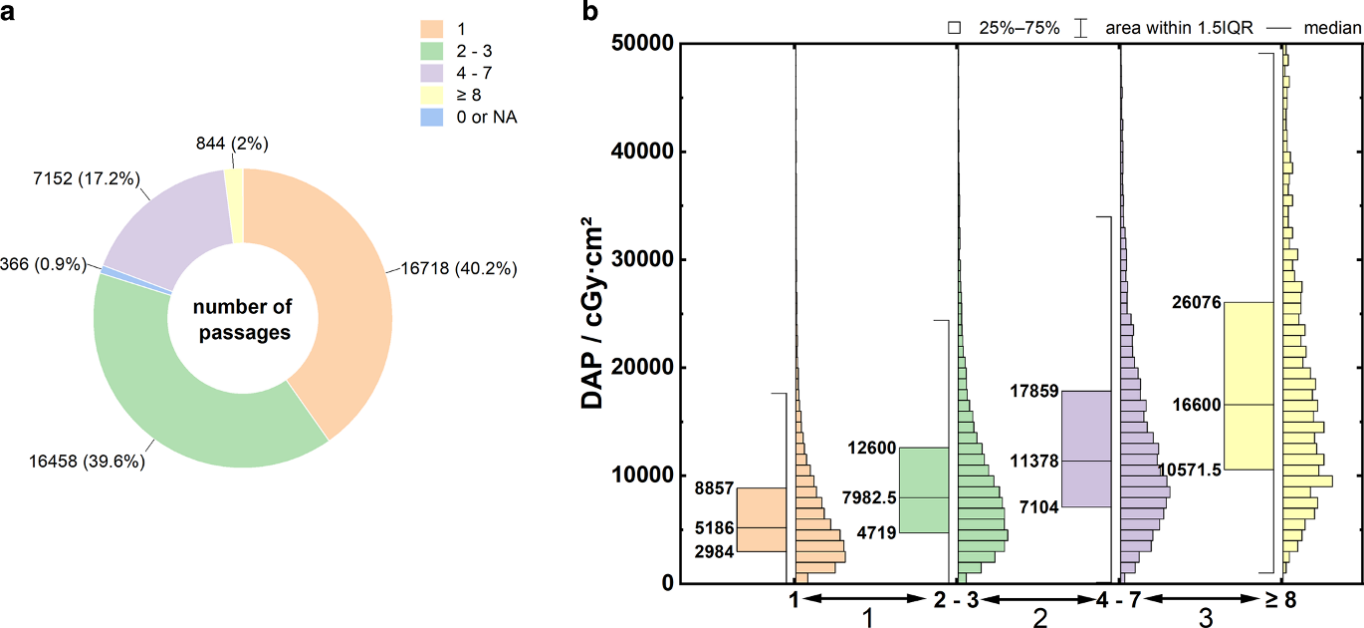
**a** Frequency distributions of the required recanalization passages during MT. **b** Boxplots of DAP distributions as a function of the number of passages required. Mood’s median test: *p* < 0.001, Dunn’s test: *p* < 0.001 (1), *p* < 0.001 (2), *p* < 0.001 (3). Specification of Q_25_, Q_50_, and Q_75_ at the box plots

Figure 6a shows the frequency distribution of the required thrombectomy passages. Most interventions (79.8%) were completed after a maximum of three passages. More than seven passages were required in only 2% of the cases. The maximum number of documented passages is 20.

Figure 6b shows the dose distributions for the DAP as a function of the required number of passages. The key parameters of the distributions are summarized in Table 3, rows 14–17. Four groups were formed to improve the statistics. Group boundaries were defined such that the median DAP increased by approximately 50% from 1 group to the next. The correlation between increased number of passages and radiation exposure was statistically significant (*p* < 0.001).

### Case Volume per Center

To investigate the influence of the performing center on patient dose, registry data were divided into three case volume groups according to the number of cases per center in the analysis period of 3 years: ≤ 150 (1), 151–450 (2), ≥ 451 (3).

**Fig. 7:**
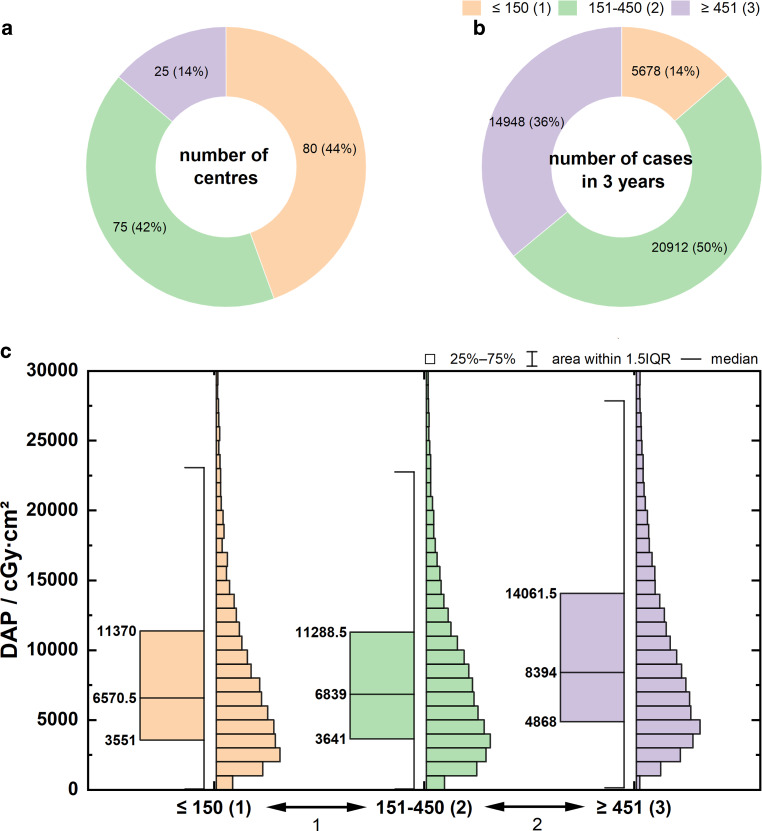
**a** Number of centers per case volume group (period: 3 years). **b** Number of registered data per case volume group (period: 3 years). **c** Boxplots of DAP distributions as a function of case volume per center *p* < 0.001, Dunn’s test: *p* = 0.3 (1), *p* < 0.001 (2). Specification of Q_25_, Q_50_, and Q_75_ at the box plots

Figure 7a shows the number of centers, and Fig. 7b shows the number of cases entered per case volume group.

Figure 7c shows the dose distributions for DAP depending on the case volume group. The key parameters of the distributions are summarized in Table 3, rows 18–20. Although no significant dose difference was detected between case volume groups 1 and 2 (*p* = 0.3), the average patient exposure in group 3 was significantly higher, by approximately 30% (*p* < 0.001).

## Discussion

To date, this is the largest study on patient dose in interventional stroke treatment. Our data showed dose dependences on the recanalization technique, additional stenting, occlusion localization, number of passages, number of cases per center, and recanalization success.

Table 4 summarizes the current comparative studies on patient exposure during MT. With the exception of the study by Klepanec et al. [14], in which only monoplanar interventions were considered, patient exposure in previous studies were higher than that observed in this study. The exact fractions of monoplanar or biplanar interventions used in this study are unknown; however, it can be assumed that most interventions were performed on biplane angio suites.

**Table 4:** Comparative studies of patient exposure during MT

Parameter	Schegerer et al. [9]	Weyland et al. [11]	Guenego [18]	Peter et al. [12]	Farah et al. [15]	Klepanec et al. [14]	Weyland et al. [13]		This study	
Data volume	< 65,000^a^	544	520	208	319	179	68	68	8290	31,026
Number of centers	< 244^a^	1	5	1	1	1	1		180	
Period	2012–2017	2013–2018	2014–2017	2017–2018	2015–2017	2017–2019	2014–2019		2019–2021	
Occlusion localization	Not specified	Not specified	Not specified	Not specified	Not specified	Not specified	Anterior circulation		Not specified	
Technical approach	Aspiration	Aspiration and/or stent retrieval	Aspiration and/or stent retrieval	Aspiration and stent retrieval	Aspiration and/or stent retrieval	Mostly aspiration	Aspiration	Stent retrieval	Aspiration	Stent retrieval
DAP median (IQR = 25–75)	9100 (5100–15,800) cGy·cm^2^	11,370 (6890–18,170) cGy·cm^2^	9100 (5700–14,800) cGy·cm^2^	8660 (NA) cGy·cm^2^	9400 (6200–16,200) cGy·cm^2^	2190 (NA–3410) cGy·cm^2^	6260 (4170–8940) cGy·cm^2^	8980 (5370–13,170) cGy·cm^2^	5280 (2880–9140) cGy·cm^2^	7984 (4538–13,021) cGy·cm^2^
Comment	Exclusion of data > 3 × DRL and < 0.1 × DRL	Only biplanar	Only biplanar with automatic exposure control	Only biplanar	Biplanar and monoplanar	Only monoplanar	Only biplanar, examiner experience > 25 procedures, no additional angioplasty required		–	–

^a^Not defined how much of the data relates to MT

In the largest comparative study by Schegerer et al. [9], MT was only one of several X‑ray applications evaluated, all data > 54,000 cGy∙cm^2^ and < 1800 cGy∙cm^2^ were excluded. If these criteria were applied to the DeGIR/DGNR data presented here, the median and IQR would increase to 7750 cGy∙cm^2^ and 4597–12,519 cGy∙cm^2^, respectively; however, they would remain below the values reported by Schegerer et al.

The decreasing trend observed with respect to patient exposure during MT may be due to the wider availability of new device technologies with automatic exposure control (AEC) and increased experience in performing MT.

### Recanalization Success

The recanalization rate of 89.7% (mTICI score of ≥ 2b) observed here is consistent with the results of the current literature, which report technical success rates of 87–90% [19, 20].

Farah et al. [15] and Klepanec et al. [14] reported significantly lower patient exposure in successful versus unsuccessful recanalization. Unsuccessful interventions were more often associated with an increased number of thrombectomy passages, averaging 3.8 (mTICI < 2b) vs. 2.3 (mTICI ≥ 2b). The lowest dose values were achieved only in rapid and successful interventions, with an mTICI of 3.

### Technical Approach

If MT is performed by stent retrieval, the patient exposure is approximately 50% higher than that by aspiration alone. As stent retrieval was used in most cases (72.1%), the dose distribution of MT was primarily characterized by stent retrieval. These results are consistent with those obtained by Weyland et al., in which a dose difference of approximately 43% was observed between stent retrieval and aspiration [13].

Because most examinations are performed using stent retrieval, the official DRL should also be based on this group. Therefore, a DRL of 14,000 cGy·cm^2^ is recommended. This also corresponds to the current DRL in Germany [10]. If the occlusion can be removed by aspiration alone, the data indicate that a lower dose of exposure can be expected.

### Additional Stenting (Extracranial/Intracranial)

When MT was performed with extracranial stenting (tandem occlusions), an increased patient exposure of almost 50% occurred on average compared with MT without additional stenting because of the higher complexity of the procedure. These results are consistent with those of Peter et al. [12], who observed dose dependence between tandem occlusions and anterior or posterior circulation occlusions alone. When intracranial stenting or a combination of extracranial and intracranial stenting, was necessary, the data showed a dose increase of 100–150% on average; however, in these cases, only a limited amount of data must be considered (see Fig. 4a).

### Occlusion Localization

The mean DAP for occlusions in multiple vascular territories was approximately 20% higher than that for occlusions in anterior or posterior circulation alone. If the occlusions of the anterior circulation were confined to the MCA, the mean DAP was 26% lower. No significant dose difference was observed between the occlusions of the anterior (ACI + ACA/MCA) and posterior circulations (*p* = 0.07), which is consistent with the results of Peter et al. [12] and Farah et al. [15].

### Number of Passages

Overall, the dose clearly increased with the number of passages, which was in agreement with previous studies [11, 12]. Compared to a single thrombectomy passage, the patient exposure doubled after 4 passages and tripled after approximately 8–9 passages.

### Case Volume per Center

A study by Weyland et al. demonstrated that the learning curve is the largest for the first 25 examinations, and that dose-dependence on examiner experience is hardly detectable after more than 25 examinations [16]. No dose-dependence on the number of cases was observed between groups 1 and 2 for the grouping chosen here. Furthermore, regarding the recanalization rate (mTCI ≥ 2b), no difference was observed between groups 1 (90.0%), 2 (89.6%), and 3 (89.8%).

However, it is surprising that patient exposure was significantly higher in group 3 at the most experienced centers. This may be attributed to the increased incidence of complicated cases and/or the increased training of residents in these high-volume centers.

Overall, the results demonstrate a consistently high level of quality in MT performance provided by various centers.

### Limitations

Entry into the DeGIR/DGNR registry is voluntary, which implies that not all hospitals participate and some entries may be incomplete or incorrect. Therefore, the validity of the data depends on the correct choice of suitable filter criteria, which can increase the bias effect. Documentation of dose data in the DeGIR/DGNR registry is limited to DAP and FT and is thus severely restricted. Previous studies have demonstrated that the disclosure of skin doses or at least the reference air kerma is necessary for the estimation of deterministic radiation damage, especially for interventional procedures such as MT [21].

## Conclusion

This study represents the largest dose evaluation for thrombectomy patients based on DeGIR/DGNR registry data. The results demonstrated a decreasing trend in patient exposure during MT in Germany. Based on the available data, the current DRL of 14,000 cGy·cm^2^ was deemed appropriate.

The recanalization technique, occlusion localization, number of required passages, and recanalization success affect radiation exposure. These factors can be used to identify the cause of excess DRL and optimize the treatment workflow.
